# A New Poison

**Published:** 1877-03

**Authors:** 


A NEW POISON.—The *Medical Press* and *Circular* says the Garden of Acclimatization of Paris has received several specimens of one of the most interesting of the flora of Rio Nunez—the Teli. The tree is an almost unknown species, and produces one of the most violent of existing poisons, which has recently been tried with success in several cases of tetanus. The Teli grows from sixty to eighty feet high. Its wood is very hard and close, and is much sought after for the building of boats, in consequence of its resistance to decay. Its bark affords a violent poison. In important lawsuits among the aborigines of the country, and when the proof of the case by evidence fails, teli is administered as an infusion, as an "ordeal" to both the parties to the cause. He who survives is declared innocent, but almost always both die. No antidote is known, but the natives combat its effects by means of the bark of the *boulle-bete*, a species of acacia which produces abundant vomiting.—*Med. and Surg. Reporter.*

